# Psychological Distance Impacts Subgroup Reciprocity in Technological Innovation Networks: The Mediating Role of Divisive Faultlines

**DOI:** 10.3389/fpsyg.2022.857088

**Published:** 2022-03-16

**Authors:** Dongping Yu, Kaixin Deng, Xiangmao Gao, Yongsong Liu

**Affiliations:** ^1^International Business School, Yunnan University of Finance and Economics, Kunming, China; ^2^International Languages and Cultures School, Yunnan University of Finance and Economics, Kunming, China

**Keywords:** psychological distance, divisive faultlines, technological innovation network, subgroup reciprocity, partner selection

## Abstract

As information flows at ever-increasing speeds across technological innovation networks, it is crucial to optimize reciprocity among partnering enterprises. However, the impact of psychological distance on subgroup reciprocity in such networks has not yet been investigated. To address this gap, the current study drew on theories of faultlines and cohesive subgroups to model the relationship between psychological distance and subgroup reciprocity within technological innovation networks. Our hypotheses were tested using data from 174 respondents working in Yunnan Province, China. The results were as follows: first, psychological distance had negative effects on subgroup reciprocity in technological innovation networks; second, relationship-divisive and innovation-divisive faultlines negatively impacted reciprocity within and between subgroups; third, the faultlines partially mediated the negative relationship between psychological distance and intra-subgroup reciprocity; and fourth, the negative relationship between psychological distance and subgroup reciprocity was not mediated by the faultlines. The findings uncover the psychological mechanism of subgroup reciprocity within technological innovation networks. They will inform the decision-making process of enterprises when selecting partners within their technological innovation networks and support the development of effective reciprocal relationships with other innovators.

## Introduction

It is a vital way for firms to optimize their core innovation resources by crossing organizational boundaries and building collaborative innovation networks with other organizations to share heterogeneous resources. These forms of networking enable organizations to expand their competitive advantages in the changing global economic landscape and the repeated intertwining of the new crown epidemic (Chin et al., 2022).

However, it is still prominent for firms in technological innovation networks to fall in traditional dilemmas such as “collaboration vs. competition” or “trust vs. suspicion” (Hoffmann et al., 2018), eventually leading the network to form several distinct, internally homogenous subgroups (Caner et al., 2017). The technological innovation network retains an overall unity while also displaying loose coupling and local fragmentation (Cheng et al., 2017). Subsequently, each innovator will build reciprocal innovative behaviors with others within the same subgroup or with those of other subgroups based on different partner selection mechanisms (i.e., combining internal cohesion and historical partner preferences to build reciprocity within or between groups, as predicted by theories of multiculturalism and heterogeneity). These mechanisms can be summarized as subgroup reciprocity in technological innovation networks.

To understand variation in the performance of innovating subjects in terms of the reciprocal behaviors of subgroups within their networks, scholars have focused on knowledge flow (Duan et al., 2021a, 2022), resource sharing (Lacerda and van den Bergh, 2020) and value creation (Chin et al., 2021) among other variables. However, such research has tended to overlook the importance of heterogeneous reciprocity behaviors that are rooted in psychological factors. Even literature that has investigated such factors (Huo and Molina, 2006) has focused on the recognition, acceptance, and valuation of members without systematically considering how these relate to subgroup reciprocity.

The theories of social embeddedness (Ren et al., 2021) and partner selection (Braun and Sydow, 2019) indicate the importance of psychological distance when selecting partners within the technological innovation network. Psychological distance refers to organizations’ subjective cognitive bias arising from various aspects such as attribute (Wang and Zhang, 2017). Traditional partner selection theory argues that firms within a technological innovation network usually follow convention when selecting reciprocal business partners, i.e., they will prefer firms that are more familiar (Acedo-Carmona and Gomila, 2015) or with whom they have previously cooperated (Wei et al., 2020). This is because greater psychological distance between reciprocal partners is more likely to impede the flow of information between them (Calabrò et al., 2021). This is not conducive to establishing and maintaining stable reciprocal relationships between innovators, since it leads to communication barriers and decreases cohesion among network members (Doucerain et al., 2015; Straube et al., 2018). Moreover, it may limit improvements in the innovators’ abilities, reducing the value co-created by reciprocal action (Zhao et al., 2015) and impacting the level of innovation that is achieved. Therefore, psychological distance may be viewed as a key antecedent of subgroup reciprocity in technological innovation networks.

The theories of faultlines (Kavuşan et al., 2020) and cohesive subgroups (Meleady et al., 2021), clarify that divisive faultlines are key antecedents to the formation of subgroups within technological innovation networks. Divisive faultlines refer to the tendency to divide within the overall network due to differences in the degree of shared experience among members of nodal organizations during interactive innovation (Dang et al., 2016; Cheng et al., 2017). Firms in the network will have some strategic risks during the process of technological innovation networks’ sustainable innovation (Duan et al., 2021b). Variations in both the attributes of innovating subjects and the conventions for selecting partners may produce divisive faultlines in technological innovation networks as they develop.

Technological innovation networks are divided into many potential subgroups of varying sizes. When divisive faultlines have been formed, they are often reflected by strong internal cohesion *within* each group and low cohesion or even conflict *between* them (Aristei et al., 2016). This differentiates each group’s ability to participate in reciprocal innovation activities and co-create value with other subjects in the network (Kapoor and Furr, 2015). Divisive faultlines therefore help to explain the role of psychological distance in intra- and inter-subgroup reciprocity.

To sum up, this study drew on a range of theories, including those of resource dependence, social embeddedness, partner selection, reciprocity, faultlines, and cohesive subgroups. These were used to construct a theoretical model of psychological distance and innovative subgroup reciprocity with divisive faultlines as an intermediary variable. We aimed to analyze the psychological mechanisms and pathways that influence this form of reciprocity to guide those responsible for selecting innovative business partners.

## Literature Review and Hypotheses

### Psychological Distance and Subgroup Reciprocity

The concept of psychological distance was first introduced by Beckerman (1956) and later modified by Vahlne and Wiedersheim-Paul (1973), who highlighted it as a vital disruptor that affects a person’s understanding of his or her external environment. Psychological distance in this paper refers to innovators’ subjective perceptions of the varying strengths of organizational reciprocity that affect the flow and sharing of innovation resources during reciprocal activities in technological innovation networks.

Subgroups are a subset of actors that have a stable, direct, solid, or frequent positive connection with each other (Knoke, 2009). They are a fundamental unit of analysis for understanding the structure of innovation networks and the embeddedness of innovating subjects within them (Shipilov et al., 2014). The study of reciprocal relations in technological innovation networks from the subgroup perspective has informed much recent research into collaborative innovation. The members of technological innovation networks maintain both contractual and practical independence from each other. They rely on the common interest of their organizations and their reciprocal actions (Jasini et al., 2018). Through a lengthy and continuous process of mutual identification and relationship building, a stable and close organizational network based on competing relationships is formed. The current study considers subgroup reciprocity in technology innovation networks as reciprocating behaviors carried out by members of these subgroups. Considering the possibility that there are differences in the antecedents and consequences when selecting technology partners within and outside the subgroups, this study creatively divides subgroup reciprocity into two dimensions (they are intra-subgroup reciprocity and inter-subgroup reciprocity) to deeply analyze the differences and causes of reciprocity behaviors intra- and inter-subgroup. Subgroup reciprocity in technological innovation networks as reciprocating behaviors carried out by members of these subgroups, i.e., enterprises, both within their own group (that is called as intra-subgroup reciprocity) and with others (that is called as inter-subgroup reciprocity).

The study analyzes the relationship between psychological distance and subgroup reciprocity from two perspectives: information flow and subjective perception. Previous findings indicate that psychological distance inversely affects subgroup reciprocity by reducing the flow of innovation resources between firms. It is an important external factor that blocks the flow of information between innovation agents (Afuah, 2013; Hutzschenreuter et al., 2014). Psychological distance between firms may lower the rate of information exchange and increase the cost of searching and acquiring information, thus limiting the benefits of subgroup reciprocity. On the other hand, it may reverse such benefits by reducing the willingness of enterprises to share innovation resources. Lavie and Khanna (2012) found that psychological distance increased organizations’ perceptions of hesitation in reciprocal interaction and hindered the establishment of intimate and stable resource-sharing relationships among firms, thereby preventing the formation of reciprocal networks characterized by rational resource allocation and close relationships. Similarly, Wang et al. (2013) argued that psychological distance reduced the network embeddedness of innovation subjects, weakened enterprises’ knowledge-sharing behavior, and thus limited the effects of reciprocal innovation. Zheng et al. (2017) argued that greater psychological distance reduced innovators’ willingness to establish reciprocal relationships and share their resources. These findings led us to formulate the following hypotheses:

*H1a*: Psychological distance is negatively associated with intra-subgroup reciprocity in technological innovation networks. That is, the greater the psychological distance, the less reciprocity exists among technological innovators in the same subgroup.

*H1b*: Psychological distance is negatively associated with inter-subgroup reciprocity in technological innovation networks. That is, the greater the psychological distance, the less reciprocity exists between subgroups of technological innovators.

### Divisive Faultlines and Subgroup Reciprocity

Divisive faultlines in technological innovation networks refer to the overall tendency toward differentiation within the network. This is caused by differences in the experience that nodal organizations share during interactive innovation (Dang et al., 2016; Cheng et al., 2017). Faultlines can be divided explicitly into *relationship-divisive* faultlines that refer to varying strengths of the relationship between innovating subjects and *innovation-divisive* faultlines that denote differences in the innovative capabilities of these subjects.

Relationship-divisive faultlines form as innovating subjects interact. On the one hand, they may cause excessive resource homogeneity within subgroups, negatively affecting the reciprocity of their members. If the technology innovation network is viewed as a rich pool of resources, the connections among members are the pipeline through which information and knowledge flow within and among subgroups (Lau and Murnighan, 2005). However, divisive faultlines in relationships arise during interaction as different cultural backgrounds, statuses, and emotions are perceived and enacted. These eventually split the network into several subgroups that are internally homogeneous and externally heterogeneous (Duysters and Lemmens, 2003). When the type, scale, technology, and other aspects of each enterprise in the subgroup are highly similar, some innovation resources in the subgroup such as shared technology, and knowledge become overly homogenous and outdated (Yan and Guan, 2018). This results in two forms of knowledge redundancy within the enterprise and its subgroups, and militates against the organizational integration of heterogeneous knowledge and the development of diverse, innovative products (Xu and Hou, 2020). Moreover, it reduces the competitive advantage of enterprises and the reciprocal effects of innovation within subgroups. It may reduce cohesion or even cause conflict between subgroups (Zhang and Guler, 2020), making inter-subgroup reciprocity harder to achieve. Relationship-divisive faultlines lead to inconsistencies within the network (Thatcher and Patel, 2012), and may produce conflicts linked to both tasks and emotions (Maltarich et al., 2021), negatively impacting team performance. Straube et al. (2018) hold a similar view, arguing that deeper relationship-divisive faultlines make smooth communication among members more difficult, and vice versa.

Innovation-divisive faultlines arise from differences in the capabilities of innovating subjects. On the one hand, they decrease the effectiveness of resources within subgroups, negatively impacting reciprocity within them. The network split caused by these faultlines homogenizes the innovative abilities of agents within subgroups while making the innovation capabilities of different groups more heterogeneous. Therefore, members may only be able to access similar external resources from other partners within the subgroup, reducing their innovative value (Moeen and Agarwal, 2017). Thus, excessive similarities among innovation subjects can curtail innovation (Cobeña et al., 2017). Innovation-divisive faultlines reduce the effectiveness of resources such as knowledge and technology within subgroups, reducing the value of reciprocity (Bouncken et al., 2020). On the other hand, these faultlines can lead to excessive differences in innovation status between subgroups, negatively affecting inter-subgroup reciprocity. Different identities, values, and codes of behavior among firms in different subgroups thereby lead to significant variations in innovation status and tensions between subgroups (Bouncken et al., 2020), reducing the efficiency of their coordinated activities (Khamseh et al., 2017). This further disconnects their approaches to innovation activities (Ferreira et al., 2021), and ultimately damages inter-subgroup reciprocity. Based on these assertions, the following hypotheses were proposed:

*H2a*: Relationship-divisive faultlines are negatively associated with intra-subgroup reciprocity in technological innovation networks; that is, the stronger the relationship-divisive faultlines, the weaker the intra-subgroup reciprocity.

*H2b*: Relationship-divisive faultlines are negatively associated with inter-subgroup reciprocity in technological innovation networks; that is, the stronger the relationship-divisive faultlines, the weaker the inter-subgroup reciprocity.

*H3a*: Innovation-divisive faultlines are negatively associated with intra-subgroup reciprocity in technological innovation networks; that is, the stronger the innovation-divisive faultlines, the weaker the intra-subgroup reciprocity.

*H3b*: Innovation-divisive faultlines are negatively associated with inter-subgroup reciprocity in technological innovation networks; that is, the stronger the innovation-divisive faultlines, the weaker the inter-subgroup reciprocity.

### Psychological Distance and Divisive Faultlines

The five major triggers of most faultlines are differential treatment, different values, assimilation, humiliating or shaming behaviors, and simple contact (Chrobot-Mason et al., 2009). Among these triggers, the antecedent role of differential treatment and different values in forming divisive faultlines has been most widely researched. Huo and Molina (2006) argued that the sensitivity of group members to divisive faultlines should be addressed by enhancing inter-organizational psychological compatibility as a means of improving group reciprocity. However, Hülsheger et al. (2009), contended that factors such as intra-group cohesion and inter-group goal interdependence can hinder the formation of productive relationship-divisive faultlines. Chrobot-Mason and Aramovich (2013) later extended this finding to the intergroup level. As a potential network structure (Mäs et al., 2013), the mechanism by which divisive network faultlines form is also related to the establishment of reciprocal partnerships. That is, the diversity of network members’ individual and relational attributes revealed during interaction may lead divisive faultlines to form in technology innovation networks (Ren et al., 2015).

To some extent, this also confirms the effects of psychological distance on the divisive faultlines in such networks. When individuals recognize slight differences in the strength of their ties and ability to innovate between themselves and their partners, they will initiate improvements in the relationship in order to reduce the occurrence of faultlines. In contrast, when such strengths and abilities differ more obviously, individual agents choose to establish reciprocal relationships with firms more similar to themselves, and both relationship-divisive and innovation-divisive faultlines are more likely to occur.

In summary, psychological factors can lead to unevenness in the strength of ties and produce relationship-divisive faultlines by affecting the establishment and maintenance of reciprocal innovation relationships between organizations. Moreover, they also produce uneven innovation capacities and innovation-divisive faultlines by affecting the development and quality of reciprocal innovation activities in organizations. Drawing on the findings of Azar and Drogendijk (2014), this study explored the relationship between psychological factors and divisive faultlines by considering psychological distance as a key antecedent in the formation of relationship and innovation-divisive faultlines. Accordingly, the following hypotheses were formulated:

*H4a*: Psychological distance is positively associated with relationship-divisive faultlines; that is, the greater the psychological distance, the stronger these faultlines will be.

*H4b*: Psychological distance is positively associated with innovation-divisive faultlines; that is, the greater the psychological distance, the stronger these faultlines will be.

### The Mediating Role of Divisive Faultlines

In technology-intensive industries, similarity and intimacy among reciprocal partners are conducive to improving the capacity of firms to continuously acquire and absorb technological knowledge from their innovation networks, maintaining their competitive advantage in fast-changing environments (Ramaswamy and Chopra, 2014). At the same time, the flow of knowledge, technology and other resources within and between subgroups in technological innovation networks enables enterprises to acquire tacit, adaptive, and innovative knowledge, thereby facilitating reciprocal innovation within the networks.

Significant psychological distance between members of technological innovation networks leads to uneven reciprocal relationships, in turn forming relationship-divisive faultlines and causing differences in reciprocal innovation within and outside the subgroup. The creation of relationship-divisive faultlines is not conducive to searching and acquiring more productive technical expertise (Zhang and Guler, 2020). This reduces the professional sensitivity of enterprises to innovative knowledge and current trends in technology while increasing the cost of acquiring innovation resources (Xu, 2015), negatively impacting both intra- and inter-subgroup reciprocity in technological innovation networks. On the other hand, psychological distance can also lead to uneven innovation capabilities within network actors, generating the innovation-divisive faultlines that underlie differences in reciprocal innovation within and outside the subgroups. These faultlines not only restrict access to diverse innovation resources within the network but also stimulate actors to imitate the innovation patterns of similar members. Ultimately, this leads to the homogenization of innovation resources and increases the cost of searching, acquiring, and integrating value-based innovation resources (Golooba and Ahlan, 2013). Therefore, both intra-subgroup and inter-subgroup reciprocity are impacted. These relationships were predicted in the following hypotheses:

*H5a*: Relationship-divisive faultlines mediate the relationship between psychological distance and intra-subgroup reciprocity in technological innovation networks.

*H5b*: Relationship-divisive faultlines mediate the relationship between psychological distance and inter-subgroup reciprocity in technological innovation networks.

*H6a*: Innovation-divisive faultlines mediate the relationship between psychological distance and intra-subgroup reciprocity in technological innovation networks.

*H6b*: Innovation divisive faultlines mediate the relationship between psychological distance and inter-subgroup reciprocity in technological innovation networks.

**Figure 1:**
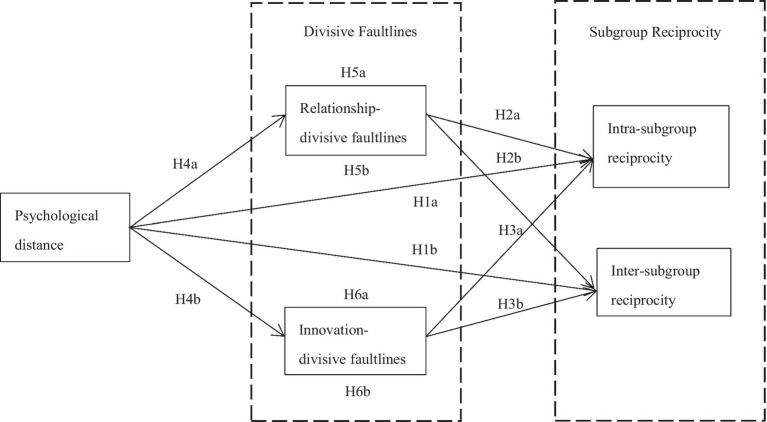
The theoretical model.

Figure 1 shows how the relationship between these variables was modeled in this study, based on hypotheses 1–6.

## Materials and Methods

### Measurement of Variables

Data on psychological distance, network-divisive faultlines, and subgroup reciprocity were collected *via* a large-scale enterprise questionnaire. We first piloted the instruments with a small sample of the study population to ensure data quality. After taking feedback from participants and conducting a factor analysis of these results, some items were revised and the survey was distributed.

Most of the items were obtained from existing scales and translated by several professional management researchers with overseas study and work experience. The first section of the questionnaire aimed to gather background information about the enterprises, including the nature of their work, their scale, their R&D activities, and their collaboration with other firms The purpose of the second section was to measure the variables involved in the practices of enterprises, including psychological distance, the two dimensions of subgroup reciprocity in the networks (intra- and inter-subgroup reciprocity), and the two dimensions of network-divisive faultlines (relationship- and innovation-divisive faultlines). A seven-point Likert scale required subjects to rate their level of agreement with the statements.

#### Explanatory Variable

The explanatory variable in this study was psychological distance (PD). The main references were Salzmann and Grasha (1991) and Li and Zhang (2018), who measured PD with six items, such as “This enterprise is distant from other firms in the technological innovation network.”

#### Explained Variable

The explained variable in this study was subgroup reciprocity (SR) in technological innovation networks. Using the instrument developed by Corsten and Kumar (2005) and Kuang and He (2020), we measured intra-subgroup reciprocity (intra-sr) using four items, such as “In the process of collaborative innovation with intra-subgroup partners, developers have acquired new technology & service knowledge.” Using four items such as “In the process of collaborative innovation with inter-subgroup partners, developers have acquired new technology & service knowledge” to measure inter-subgroup reciprocity (inter-sr).

#### Intermediary Variable

The intermediary variable in this study was the divisive faultlines (denoted by DF). Referring mainly to Castro and Roldán (2015) and Wang and Guo (2020), we measured relationship divisive faultlines (df_r) using three items; for example, “There is no sound collaboration mechanism developed between enterprises in the technological innovation network.” Similarly, three items were used to measure innovation-divisive faultlines (df_i); for instance, “The degree of innovation knowledge between enterprises in the technological innovation network is very different.”

#### Control Variables

We considered four potential factors impacting reciprocity: the enterprise’s scale (Noori et al., 2017), the nature of its work (Li and Cai, 2015), its R&D activities (Brockman et al., 2018), and its collaboration with other firms. The scale of each enterprise was categorized according to the size of its workforce (1 = less than 500 employees, 2 = 501–1,000, and 3 = more than 1,000). The nature of each enterprise was described as follows: 1 = state-owned enterprise, 2 = private enterprise, 3 = others. The average R&D budgets of the enterprises over the past 3 years was denoted as 1 = less than 3%, 2 = 3–10%, 3 = more than 10%. Finally, collaboration was measured by the maximum length of collaboration with another institution, 1 = less than 5 years, 2 = more than 5 years. These factors were treated as control variables in the statistical analysis.

### Description of The Sample

Data such as psychological distance, network divisive faultlines, and subgroup reciprocity are not available from public information. So this study used a large-scale enterprise questionnaire for data collection and empirical analysis. We did a small sample data collection and pre-test to ensure data quality. After that, the questionnaire was reasonably revised, and then a large-scale questionnaire was distributed.

It is very famous to consider the number of collaborative patent applications to measure the effectiveness of reciprocal innovation among enterprises (Breschi and Lenzi, 2016; de Araújo et al., 2019) and the convenience of sample collection in the region where our team is located. So this study first searched the data of enterprise collaboration patents registered in the Yunnan province during 2010–2021 through the patent search system of China’s State Intellectual Property Office, and a total of 39,975 data were retrieved. Secondly, each enterprise’s number of collaborative patents during the search period was counted and arranged in descending order. Then, based on the research of Pereira et al. (2018), the top 250 enterprises were selected as the questionnaire objectives. Finally, with the help of the Yunnan Provincial Department of Science and Technology, one questionnaire was distributed to each sample enterprise mainly by field distribution and supplemented by mail and telephone distribution. The top manager for each enterprise was asked to fill it out. Ultimately, 192 of the 234 surveys we distributed were returned for 5 months, a recovery rate of 82.1%. Of these, 18 incomplete questionnaires were removed, leaving 174 usable, an overall efficiency rate of 74.3%, which is higher than the one of 6.8–11% in behavioral studies by Hong Kong scholars and the one of 10–33% in empirical studies by Western scholars (Man et al., 2002), indicating that the questionnaire return rate is within an acceptable range.

Table 1 shows the descriptive statistics for the sample. In terms of the scale of the enterprises, 46.6% had less than 500 employees, 16.1% had 501–1,000 employees, and 37.4% employed over 1,000 workers. State-owned enterprises accounted for 65.5% of the sample, private enterprises for 25.3%, and others, 9.2%. For average R&D intensity over the past 3 years, the results indicated that 30.5% of the enterprises had invested 3% of their budgets in R&D, 43.7% had invested between 3 and 10%, and 25.9% had invested over 10%. Finally, 47.1% of the sample reported a maximum collaboration length of below 5 years while 52.9% had collaborated with at least one partner for more than 5 years.

**Table 1:** Descriptive statistics for the sample.

Characteristic	Classification	Frequency	Percentage (%)
Size	<500 employees	81	46.6
	501–1,000 employees	28	16.1
	>1,000 employees	65	37.4
Nature	State-owned enterprises	114	65.5
	Private enterprise	44	25.3
	Others	16	9.2
R&D (The average R&D intensity of the enterprise in the past 3 years)	<3%	53	30.5
	3–10%	76	43.7
	>10%	45	25.9
Collaboration (The most extended duration of collaboration with other institutions)	<5 years	82	47.1
	>5 years	92	52.9

### Testing for Reliability and Validity

Cronbach’s *α* coefficient was used to measure the internal consistency of the factors. The coefficients for psychological distance, relationship-divisive faultlines, innovation-divisive faultlines, intra-subgroup reciprocity, and inter-subgroup reciprocity were 0.84, 0.81, 0.79, 0.95, and 0.94, respectively. These results indicate that the items had good internal consistency and point to the reliability of the scale.

An exploratory factor analysis of the samples was conducted using KMO test analysis and Bartlett’s test of sphericity. The KMO values for psychological distance, relationship-divisive faultlines, innovation-divisive faultlines, intra-subgroup reciprocity, and inter-subgroup reciprocity were 0.73, 0.70, 0.69, 0.84, and 0.84, respectively, all greater than the threshold value of 0.7. Bartlett’s spherical test of the same variables recorded scores of 0.000 for all, less than the 0.001 threshold and indicating the suitability of the data for factor analysis.

Standardized factor loadings, average variance extracted values (AVE), and combined reliability (CR) measurements were used to gage the convergent validity of the sample. As Table 2 shows, the factor loading coefficients of all items used in the questionnaire exceeded the threshold value of 0.7. Similarly, the AVE and CR values (at least 0.62 and 0.83, respectively) indicated the high convergent validity of the scale used in the study.

**Table 2:** Tests of reliability and validity.

Variable		Number	Factor loading	Cronbach’s *α*	KMO	Bartlett	AVE	CR
Psychological distance (PD)		PD1	0.769	0.839	0.725	0.000	0.773	0.953
		PD2	0.905					
		PD3	0.877					
		PD4	0.865					
		PD5	0.933					
		PD6	0.918					
Divisive faultlines (DF)	Relationship-divisive faultlines (df_r)	DF1	0.774	0.807	0.836	0.000	0.635	0.839
		DF2	0.847					
		DF3	0.767					
	Innovation-divisive faultlines (df_i)	DF4	0.820	0.793	0.704	0.000	0.619	0.830
		DF5	0.766					
		DF6	0.773					
Subgroup reciprocity (SR)	Intra-subgroup reciprocity (intra-sr)	SR1	0.859	0.947	0.694	0.000	0.750	0.923
		SR2	0.893					
		SR3	0.877					
		SR4	0.835					
	Inter-subgroup reciprocity (inter-sr)	SR5	0.825	0.942	0.835	0.000	0.733	0.916
		SR6	0.828					
		SR7	0.881					
		SR8	0.888					

A validated factor analysis was conducted for each variable using SPSSAU software to test for discriminant validity. The AVE square root value for each of the five variables was greater than the maximum correlation coefficient values shown in Table 3 (0.754), indicating good discriminant validity among the variables in this study.

**Table 3:** Correlation analysis of the variables.

	PD	df_r	df_i	Intra-sr	Inter-sr	Size	Nature	R&D	Collaboration
PD	1								
df_r	0.657^***^	1							
df_i	0.676^***^	0.754^***^	1						
intra-sr	−0.567^***^	−0.481^***^	−0.534^***^	1					
inter-sr	−0.372^***^	−0.316^***^	−0.319^***^	0.683^***^	1				
Size	−0.068	−0.084	−0.006	0.012	−0.059	1			
Nature	−0.093	−0.076	−0.093	0.153^**^	0.151^**^	−0.087	1		
R&D	−0.257^**^	−0.222^**^	−0.140^*^	0.206^**^	0.200^**^	0.305^***^	0.123	1	
Collaboration	−0.241^**^	−0.207^**^	−0.182^**^	0.200^**^	0.153^**^	0.533^***^	−0.030	0.400^***^	1

* Significant at *p* < 0.1.

** Significant at *p* < 0.05.

*** Significant at *p* < 0.001.

The constant terms are omitted.

The correlations between the variables were calculated using the Pearson correlation coefficient matrix. As Table 3 shows, psychological distance had a significant negative impact on intra-subgroup and inter-subgroup reciprocity, thus tentatively validating H1 and rejecting H2. Both types of faultlines negatively impacted intra- and inter-subgroup reciprocity to a significant extent, thereby tentatively rejecting H3 and H4 and validating H5 and H6. Psychological distance had a significant positive impact on both types of faultlines, meaning that H9 and H10 were tentatively validated, while H7 and H8 were not.

## Results

### Testing of Hypotheses

To eliminate potential multicollinearity among the variables, a principal component analysis of the factors of psychological distance (PD), divisive faultlines (DF), and technological innovation network subgroup reciprocity (SR) was conducted and the underlying data was used to test each hypothesized relationship.

To test the relationship between PD and SR, a hierarchical regression analysis was conducted. First, four control variables; i.e., the enterprises’ scale (size), nature (nature), R&D budgets (R&D), and collaboration with other enterprises (collaboration), were assigned as independent variables. Intra- (intra-sr) and inter-subgroup reciprocity (inter-sr) were the dependent variables used to construct Models 1 and 2 (see Table 4). Models 3 and Model 4 in Table 4 were then built by adding PD as an independent variable. Comparing the regression results for Models 1 and 3, then Models 2 and 4, it was clear that after controlling for the four variables as described, PD exerted a significant negative effect on intra-subgroup and inter-subgroup reciprocity (*β* = −0.530 and − 0.324, respectively, at *p* < 0.001), so both H1a and H1b were supported.

**Table 4:** Results of the direct relationship between psychological distance, divisive faultlines and technological innovation network subgroup reciprocity (*N* = 174).

Variable	Model 1	Model 2	Model 3	Model 4	Model 5	Model 6	Model 7	Model 8
	Intra-sr	Inter-sr	Intra-sr	Inter-sr	Intra-sr	Inter-sr	Intra-sr	Inter-sr
Size	−0.184	−0.280^**^	−0.112	−0.236^**^	−0.154	−0.261^**^	−0.099	−0.233^**^
Nature	0.241^*^	0.224	0.176	0.184	0.196	0.196	0.170	0.185
R&D	0.233^*^	0.279^**^	0.069	0.179	0.118	0.208	0.168	0.243^*^
Collaboration	0.349^**^	0.322^**^	0.163	0.209	0.231^*^	0.249^*^	0.177	0.228
PD			−0.530^***^	−0.324^***^				
df_r					−0.378^***^	−0.233^***^		
df_i							−0.430^***^	−0.237^***^
*F*	4.275^**^	4.396^**^	17.475^***^	7.574^***^	12.365^***^	6.355^***^	15.749^***^	6.420^***^
*R* ^2^	0.092	0.094	0.342	0.184	0.269	0.159	0.319	0.160
Adj-*R*^2^	0.070	0.073	0.323	0.160	0.247	0.134	0.299	0.135

* Significant at *p* < 0.1.

** Significant at *p* < 0.05.

*** Significant at *p* < 0.001.

The constant terms are omitted.

To test the relationship between DF and SR, based on Model 1, relationship-divisive faultlines (df_r) and innovation-divisive faultlines (df-i) were added as independent variables to construct Models 5 and 7. Based on Model 2, both types of faultlines were added as independent variables to construct Models 6 and 8 (see Table 4). The Models 5 and 6 regression showed that, after controlling for the four variables (size, nature, R&D, and collaboration), relationship-divisive faultlines (df_r) had a significant negative impact on intra-subgroup (intra-sr) and inter-subgroup reciprocity (inter-sr; *β* = −0.378 and − 0.233, respectively, both values of *p* < 0.001), so both H2a and H2b were supported. The regression results of Models 7 and 8 indicated that (once the same four variables were controlled for) the innovation-divisive faultlines imparted a significant and negative effect on intra-subgroup reciprocity and inter-subgroup reciprocity (*β* = −0.430 and − 0.237, respectively, both values of *p* < 0.001), thus verifying hypotheses H3a and b.

Next, the relationship between psychological distance (PD) and divisive faultlines (DF) was tested. Model 9 was constructed by first assigning enterprise scale (size), nature (nature), R&D intensity (R&D), and collaboration length (collaboration) as independent variables, then relationship- and innovation-divisive faultlines were added as dependent variables to produce Model 10 (see Table 5). Next, PD was added as an independent variable to these two models to produce Models 11 and 12. Table 5 displays the results of this process in full. After the four variables above were controlled for, PD was found to impart a significant positive effect on both DF and df_i (*β* = 0.740 and 0.772, respectively; both values *p* < 0.001), thereby verifying hypotheses H4a and b.

**Table 5:** The mediating effects of divisive faultlines (*N* = 174).

Variable	Model 9	Model 10	Model 11	Model 12	Model 13	Model 14	Model 15	Model 16
	df_r	df_i	df_r	df_i	Inter-sr	Intra-sr	Inter-sr	Intra-sr
Size	0.079	0.197	−0.022	0.092	−0.115	−0.238^**^	−0.090	−0.227^**^
Nature	−0.121	−0.167	−0.029	−0.071	0.171	0.181	0.159	0.177
R&D	−0.304^*^	−0.152	−0.076	0.087	0.057	0.171	0.090	0.187
Collaboration	−0.313^*^	−0.401^**^	−0.054	−0.130	0.155	0.204	0.132	0.196
PD			0.740^***^	0.772^***^	−0.413^***^	−0.249^**^	−0.349^***^	−0.248^**^
df_r					−0.159^**^	−0.101		
df_i							−0.235^**^	−0.098
*F*	3.245^**^	2.613^**^	26.036^***^	29.070^***^	15.728^***^	6.584^***^	17.119^***^	6.547^***^
*R* ^2^	0.071	0.058	0.437	0.464	0.361	0.191	0.381	0.190
Adj-*R*^2^	0.049	0.036	0.420	0.448	0.338	0.162	0.359	0.161

* Significant at *p* < 0.1.

** Significant at *p* < 0.05.

*** Significant at *p* < 0.001.

The constant terms are omitted.

To Models 3 and 4, relationship-divisive faultlines (df_r) and innovative divisive faultlines (df_i) were added as independent variables to produce Models 13–16 (see Table 5). The regression results of Models 13 and 15 demonstrate that, after controlling for the four variables of enterprise size, nature, R&D intensity and collaboration, the negative effect of PD on intra-subgroup reciprocity (intra-sr) was significantly weaker compared to the results of Model 3 (*β* = −0.530, *p* < 0.001). Similarly, the negative effects of relationship-divisive (df_r) and innovation-divisive faultlines (df_i) on intra-sr were significantly weaker (*β* = −0.413 and −0.349, respectively, *p* < 0.001). Finally, both df_r and df_i exerted significant negative effects on intra-sr (*β* = −0.159 and −0.235, respectively, *p* < 0.05), indicating that both types of faultlines partially mediated the relationship between psychological distance and intra-subgroup reciprocity, thereby validating hypotheses H5a and H6a. The regression results of Model 14 and Model 16 show that after controlling for the variables of enterprise size, nature, R&D, and collaboration, the effects of both types of faultlines (df_r and df_i) on inter-subgroup reciprocity were not significant (*β* = −0.101 and − 0.098, respectively, *p* > 0.1). This indicates that neither type of faultline mediated the relationship between psychological distance and inter-subgroup reciprocity, so hypothesis H5a and H6b were not supported.

The possible explanation for the result that “H5b, H6b were not supported” are as follows. Firstly, the faultlines in this sample were insufficiently pronounced to exert any effect. The Model 14 and Model 16 regressions in Table 5 show that, while neither faultline significantly mediated the relationship between PD and inter-sr, the effects of df_r were stronger than those of df_i, as suggested by their mean scores in the sample data (3.309 and 3.080, respectively). This suggests, in part, that psychological distance (PD) may require pronounced divisive faultlines to indirectly affect the reciprocal relationships between subgroups, confirming the findings of Boyraz (2019), Zhelyazkov (2018), and other scholars.

Secondly, the size of enterprises may interfere with the mediating effect of the divisive faultlines (DF) on the relationship between PD and inter-sr. From the coefficients of the size of enterprises in Table 5, it is significant only in Model 14 and Model 16, in which the dependent variable is inter-sr. Coincidentally, neither faultline (df_r and df_i) significantly mediated the relationship between PD and inter-sr in both models. Therefore, there is the possibility that the size of enterprises interferes with the role of divisive faultlines (DF) between PD and inter-sr. Of course, this needs to be verified in the future.

### Robustness Test

To test the robustness of the study, the sample data were grouped to verify the hypotheses according to the variable of collaboration. Based on the robustness testing in previous studies such as Jiratchayut and Bumrungsup (2019) and Stephens and Marder (2019), this study did the robustness test by comparing the empirical results based on different sample data (the overall sample and the grouped samples divided according to the cooperation situation). Specifically, the sample data were grouped according to the variable of collaboration. Group 1 represented enterprises with a maximum duration of collaboration with other institutions of less than 5 years; Group 2, those enterprises who had collaborated for more than 5 years. The regression analysis results based on data from these two sample groups were as follows.

Tables 6 and 7 display the regression results based on the Group 1 data, which were consistent with those of the total sample (see Tables 4 and 5) except for hypothesis H5a, which was not supported (*β* = −0.094, value of *p* > 0.01). One possible explanation is that reciprocity between the Group 1 firms had not been fully established, meaning that strong relationship-divisive faultlines had not been formed and therefore reducing the significance of df_r as a mediator. Compared to the mean values of 3.309 for the total sample and 3.322 for the Group 2 sample, the mean Group 1 value of 3.062 for df_r in the technological innovation network was relatively low, indicating that relationship-divisive faultlines (df_r) may be less pronounced earlier in the process of establishing reciprocal relationships. The impact of these faultlines on psychological distance and intra-subgroup reciprocity was also limited at this early stage. This finding corresponds to those of Chung et al. (2020), Calabrò et al. (2021), and other scholars.

**Table 6:** The direct relationship between psychological distance, divisive faultlines, and subgroup reciprocity in the technological innovation network (Group 1; *N* = 82).

Variable	Model 17	Model 18	Model 19	Model 20	Model 21	Model 22	Model 23	Model 24
	Intra-sr	Inter-sr	Intra-sr	Inter-sr	Intra-sr	Inter-sr	Intra-sr	Inter-sr
Size	−0.146	−0.150	−0.081	−0.108	−0.093	−0.119	0.036	0.523^**^
Nature	0.323	0.527^**^	0.220	0.458^**^	0.229	0.470^**^	0.317^*^	−0.047
R&D	0.256	0.242	0.093	0.134	0.238	0.231	0.153	0.183
PD			−0.585^***^	−0.389^***^				
df_r					−0.392^***^	−0.236^**^		
df_i							−0.493^***^	−0.280^**^
*F*	1.837	3.821^**^	13.064^***^	7.692^***^	6.300^***^	4.718^**^	9.942^***^	5.481^**^
*R* ^2^	0.066	0.128	0.404	0.286	0.247	0.197	0.341	0.222
Adj-*R*^2^	0.030	0.095	0.373	0.248	0.207	0.155	0.306	0.181

* Significant at *p* < 0.1.

** Significant at *p* < 0.05.

*** Significant at *p* < 0.001.

The constant terms are omitted.

**Table 7:** The mediation effects of the divisive faultlines (Group 1; *N* = 82).

Variable	Model 25	Model 26	Model 27	Model 28	Model 29	Model 30	Model 31	Model 32
	df_r	df_i	df_r	df_i	Intra-sr	Inter-sr	Intra-sr	Inter-sr
Size	0.134	0.369^*^	0.059	0.284^*^	−0.076	−0.106	−0.023	−0.097
Nature	−0.241	−0.013	−0.122	0.123	0.208	0.455^**^	0.245	0.463^**^
R&D	−0.045	−0.208	0.144	0.007	0.107	0.137	0.095	0.134
PD			0.679^***^	0.773^***^	−0.521^***^	−0.374^**^	−0.427^**^	−0.360^**^
df_r					−0.094	−0.022		
df_i							−0.204^*^	−0.036
*F*	0.585	1.183	12.921^***^	22.914^***^	10.593^***^	6.085^***^	11.315^***^	5.481^**^
*R* ^2^	0.022	0.044	0.402	0.543	0.411	0.286	0.427	0.222
Adj-*R*^2^	−0.016	0.007	0.371	0.520	0.372	0.239	0.389	0.181

* Significant at *p* < 0.1.

** Significant at *p* < 0.05.

*** Significant at *p* < 0.001.

The constant terms are omitted.

Tables 8 and 9 show the regression results for the Group 2 sample data. The results are entirely consistent with those derived from the overall sample (see Tables 4 and 5) and therefore support the robustness of the data.

**Table 8:** The direct relationship between psychological distance, divisive faultlines, and subgroup reciprocity in the technological innovation network (Group 2; *N* = 92).

Variable	Model 33	Model 34	Model 35	Model 36	Model 37	Model 38	Model 39	Model 40
	Intra-sr	Inter-sr	Intra-sr	Inter-sr	Intra-sr	Inter-sr	Intra-sr	Inter-sr
Size	−0.142	−0.316^*^	−0.105	−0.295^*^	−0.143	−0.316^**^	−0.123	−0.305^*^
Nature	0.168	−0.005	0.130	−0.025	0.152	−0.015	0.063	−0.065
R&D	0.288	0.325^*^	0.084	0.212	0.075	0.194	0.195	0.272
PD			−0.496^***^	−0.274^**^				
df_r					−0.390^***^	−0.241^**^		
df_i							−0.410^***^	−0.237^**^
*F*	1.504	2.118	7.772^***^	3.122^**^	7.094^***^	3.365^**^	8.384^***^	3.409^**^
*R* ^2^	0.049	0.067	0.263	0.126	0.246	0.134	0.278	0.136
Adj-*R*^2^	0.016	0.036	0.229	0.085	0.211	0.094	0.245	0.096

* Significant at *p* < 0.1.

** Significant at *p* < 0.05.

*** Significant at *p* < 0.001.

The constant terms are omitted.

**Table 9:** The mediation effects of divisive faultlines (Group 2; *N* = 92).

Variable	Model 41	Model 42	Model 43	Model 44	Model 45	Model 46	Model 47	Model 48
	df_r	df_i	df_r	df_i	Intra-sr	Inter-sr	Intra-sr	Inter-sr
Size	−0.003	0.047	−0.061	−0.011	−0.118	−0.305^*^	−0.108	−0.297^*^
Nature	−0.041	−0.255	0.018	−0.196	0.134	−0.023	0.078	−0.058
R&D	−0.546	−0.226	−0.225	0.095	0.034	0.176	0.109	0.228
PD			0.780^***^	0.779^***^	−0.324^**^	−0.147	−0.288^**^	−0.146
df_r					−0.220^**^	−0.164		
df_i							−0.266^**^	−0.165
*F*	2.414^*^	0.918	18.041^***^	14.919^***^	7.383^***^	2.889^**^	8.189^***^	2.931^**^
*R* ^2^	0.076	0.030	0.453	0.407	0.300	0.144	0.323	0.146
Adj-*R*^2^	0.045	−0.003	0.428	0.380	0.260	0.094	0.283	0.096

* Significant at *p* < 0.1.

** Significant at *p* < 0.05.

*** Significant at *p* < 0.001.

The constant terms are omitted.

Overall, the test results and interpretation of the sub-samples demonstrate the robustness of the study’s conclusions (Table 10).

**Table 10:** Results of the hypothesis tests.

Hypothesis number	Hypothesis	Result
H1a	Psychological distance is negatively associated with intra-subgroup reciprocity in technological innovation networks. That is, the greater the psychological distance, the less reciprocity exists among technological innovators in the same subgroup.	Supported
H1b	Psychological distance is negatively associated with inter-subgroup reciprocity in technological innovation networks. That is, the greater the psychological distance, the less reciprocity exists between subgroups of technological innovators.	Supported
H2a	Relationship-divisive faultlines are negatively associated with intra-subgroup reciprocity in technological innovation networks; that is, the stronger the relationship-divisive faultlines, the weaker the intra-subgroup reciprocity.	Supported
H2b	Relationship-divisive faultlines are negatively associated with inter-subgroup reciprocity in technological innovation networks; that is, the stronger the relationship-divisive faultlines, the weaker the inter-subgroup reciprocity.	Supported
H3a	Innovation-divisive faultlines are negatively associated with intra-subgroup reciprocity in technological innovation networks; that is, the stronger the innovation-divisive faultlines, the weaker the intra-subgroup reciprocity.	Supported
H3b	Innovation-divisive faultlines are negatively associated with inter-subgroup reciprocity in technological innovation networks; that is, the stronger the innovation divisive faultlines, the weaker the inter-subgroup reciprocity.	Supported
H4a	Psychological distance is positively associated with relationship-divisive faultlines; that is, the greater the psychological distance, the stronger these faultlines will be.	Supported
H4b	Psychological distance is positively associated with innovation-divisive faultlines; that is, the greater the psychological distance, the stronger these faultlines will be.	Supported
H5a	Relationship-divisive faultlines mediate the relationship between psychological distance and intra-subgroup reciprocity in technological innovation networks.	Supported
H5b	Relationship-divisive faultlines mediate the relationship between psychological distance and inter-subgroup reciprocity in technological innovation networks.	Supported
H6a	Innovation-divisive faultlines mediate the relationship between psychological distance and intra-subgroup reciprocity in technological innovation networks.	Not supported
H6b	Innovation-divisive faultlines mediate the relationship between psychological distance and inter-subgroup reciprocity in technological innovation networks.	Not supported

## Conclusion and Discussion

### Conclusion

By integrating the theories of resource dependence, social embeddedness, partner selection, reciprocity, faultlines, and cohesive subgroups, this innovative study constructed a hypothetical model of the relationship between psychological distance and subgroup reciprocity in technological innovation networks, considering the mediating role of divisive faultlines. The empirical research and robustness tests of the proposed model were based on questionnaire data from 174 respondents and support the following conclusions:

First, psychological distance exerted a significant negative effect on subgroup reciprocity (both intra-subgroup and inter-subgroup reciprocity) in the technological innovation network—but its effect on intra-subgroup reciprocity was significantly higher. Comparing the regression coefficients of Model 3 and Model 4 in Table 4, the direct effect of psychological distance on intra-subgroup reciprocity (*β* = −0.530, *p* < 0.001) was significantly greater than its direct effect on inter-subgroup reciprocity (*β* = −0.324, *p* < 0.001). Moreover, the regression coefficients of Models 13–16 in Table 5 demonstrate that even when the mediating role of divisive faultlines is considered, the effect of psychological distance on intra-subgroup reciprocity in the technological innovation networks (*β* = −0.413 and −0.349, respectively, both values of *p* < 0.001) was significantly higher than the effect of PD on inter-subgroup reciprocity (*β* = −0.249 and −0.248, respectively, both values of *p* < 0.05).

Second, both types of divisive faultlines carried a significant negative effect on subgroup reciprocity (both intra- and inter-subgroup). This was significantly larger on intra-subgroup than inter-subgroup reciprocity. The regression coefficients of Models 5–8 in Table 4 demonstrate that the direct effect of divisive faultlines (whether relationship-divisive or innovation-divisive) on intra-subgroup reciprocity (*β* = −0.378 and −0.430, respectively, both values of *p* < 0.001) was significantly larger than on inter-subgroup reciprocity (*β* = −0.233 and −0.237, respectively, both values of *p* <0.001). In addition, the regression coefficients of Models 13–16 clarify that even accounting for their dual effects with psychological distance, divisive faultlines had a significantly larger effect on intra-subgroup reciprocity (*β* = −0.159 and −0.235, respectively, both values of *p* < 0.05) than relationship divisive faultlines had on inter-subgroup reciprocity (*β* = −0.101 and − 0.098, respectively, both values of *p* > 0.1).

Third, there was a significant positive effect of psychological distance on both types of divisive faultlines. In the overall sample, the regression coefficients of Models 11 and 12 in Table 5 demonstrated the significant positive effect of psychological distance on all faultlines (*β* = 0.740 and 0.772, respectively, both values of *p* < 0.001), and this was also true of the subgroup sample, as Models 27 and 28 (Table 7), and Models 43 and 44 (Table 9) demonstrate.

Fourth, divisive faultlines of both types partially mediated the relationship between psychological distance and intra-subgroup (but not inter-subgroup) reciprocity. As Table 5 shows, this is demonstrated by Models 13 and 15 for the former relationship (*β* = −0.159 and −0.235, respectively, both values of *p* < 0.05) and Models 14 and 16 for the latter (*β* = −0.10 and −0.098, respectively, both values of *p* > 0.1). In the subgroup sample, this conclusion still holds, as evidenced by the significance of the corresponding model review coefficients in Tables 7 and 9.

### Theoretical Implications

Several theoretical implications arise from these findings. First, the study found that psychological distance exerted different effects on intra-subgroup vs. inter-subgroup reciprocity in technological innovation networks. Most previous studies on these forms of reciprocity have focused on knowledge flows (e.g., Rouyre and Fernandez, 2019; Lee et al., 2020), value co-creation (e.g., Adner, 2017; Ketonen-Oksi and Valkokari, 2019) while neglecting deep psychological perspectives on reciprocity. However, drawing on prior research by Azar and Drogendijk (2014) and Wang and Zhang (2017), this study analyzed the contribution of psychological distance to the mechanism involved in intra- and inter-subgroup reciprocity in technological innovation networks. Psychological distance was found to impart a significant negative effect on subgroup reciprocity, validating results from earlier research (Wang and Zhang, 2017). Furthermore, the effect of psychological distance on intra-subgroup reciprocity was found to be significantly greater than its effect on inter-subgroup reciprocity. This novel finding represents an important contribution to literature about the influence of subgroup reciprocity in technological innovation networks, and also confirms that it is significant to divide subgroup reciprocity into intra-subgroup reciprocity and inter-subgroup reciprocity.

Second, this study explored the direct effects of divisive faultlines on subgroup reciprocity in technological innovation networks, finding that intra- and inter-subgroup reciprocity were differentially affected. Most previous empirical studies have focused on the influence of team faultlines on innovation performance (Calabrò et al., 2021; Maltarich et al., 2021), but neglected similar phenomena at the network level. Drawing on Ren et al. (2015) and Zhang and Guler (2020), this study has extended the concept of team faultlines to the network level by looking at inter-subgroup reciprocity in innovation networks. The final result demonstrated that both relationship-divisive and innovation-divisive faultlines exerted significant negative effects on intra- and inter-subgroup reciprocity, corroborating the findings of Heidl et al. (2014), and other scholars. However, in contrast to earlier research, divisive faultlines were found to affect intra-subgroup reciprocity much more than inter-subgroup reciprocity. Moreover, innovation-divisive faultlines had a greater influence on subgroup reciprocity than those that were relationship divisive. These results point to the need to study the relationship between divisive faultlines and subgroups in technological innovation networks.

Third, this study investigated the mechanism by which divisive faultlines mediate the reciprocal relationship between psychological distance and technology network subgroups. Previous studies have emphasized how divisive faultlines directly impact innovation performance (Zhang et al., 2017) while overlooking their other possible mechanisms of action. This study drew on theories of divisive faultlines and cohesive subgroups to model and verify the relationship between psychological distance and subgroup reciprocity as mediated by divisive faultlines. Psychological distance was shown to indirectly impact subgroup reciprocity in technological innovation networks *via* divisive faultlines, thereby revealing the mechanism of influence for future research.

### Managerial Implications

There are several practical implications of these findings. First, they point to the need to promote the interdependence of network actors and shrink the psychological distance between reciprocal partners. Because psychological distance has a significant negative effect on intra- and inter-subgroup reciprocity, it can weaken the awareness and depth of resource-sharing among innovators and thus limit reciprocity within the whole network. For this reason, a positive, symbiotic atmosphere must be established and encouraged in order to foster the willingness to share knowledge, information, technology, and other resources with network members, i.e., to collaborate. First, enterprises can establish online information-sharing platforms to facilitate the collection, sorting, transmission, and sharing of information, along with other aspects. Second, they can hold regular meetings to discuss innovation problems. By optimizing sharing methods and encouraging different forms of business interaction such as seminars, communication barriers and potential conflicts among members can be eased. Thus, a friendly atmosphere of mutual reliance, sharing, and collaboration can all reduce the psychological distance between reciprocal partners.

Second, it is recommended to strengthen network relationship management to mitigate the impact of network-divisive faultlines. As mentioned above, these faultlines exert a considerable negative influence on subgroup reciprocity in technological innovation networks. Moreover, it also mediates the influence of psychological distance on reciprocal actions between subgroups. Therefore, network builders should periodically review the extent to which divisive faultlines are present on their network and evaluate the degree of divisive. They should also monitor the reciprocal relationships between firms inside and outside the subgroups, intervening when required to balance the strength of relationships among network members. This will help alleviate any uneven psychological distance between members, thereby supporting the quality and sustainability of the network.

Third, a mechanism to promote inter-subgroup communication should be developed to expand the overall effect of subgroup reciprocity. As the present study has shown, psychological distance and divisive faultlines impact intra-subgroup reciprocity more significantly than inter-subgroup reciprocity. For this reason, enterprises in different subgroups should strengthen their exchange of information to maintain overall network connectivity. Enterprises should seek to expand their network of reciprocal partners and attend carefully to the exchange and sharing of complementary and heterogeneous innovation resources to build new reciprocal relationships with members outside the subgroups. They can broaden the communication channels they use with external organizations and create demand-oriented online interactive forums for accessible communication, for instance. Through such inter-subgroup communication mechanisms, members can improve their knowledge and cognition by exchanging their experiences, thereby expanding the overall effect of subgroup reciprocity.

### Limitations and Future Research

Alongside its contributions, two of the study’s limitations must be mentioned. First, the generalizability of the results was limited by the decision to focus on a single area, that of Yunnan Province, China. While this decision addressed the complexity of sampling different regions (or industries in the same region), it is consequentially impossible to claim that the findings apply to all contexts. Second, the hypothesized relationships between the variables of psychological distance, and divisive faultlines, were based on the available literature, which shaped the data that was gathered. Therefore, researchers are encouraged to explore other potential relationships or effects that may exist among the three variables.

## Data Availability Statement

The raw data supporting the conclusions of this article will be made available by the authors, without undue reservation.

## Ethics Statement

Ethical review and approval was not required for the study on human participants in accordance with the local legislation and institutional requirements. Written informed consent for participation was not required for this study in accordance with the national legislation and the institutional requirements.

## Author Contributions

YDP and KDX contributed to building the theory and revising the paper. GXM contributed to the language style and checking the quality of the paper. LYS contributed by collecting data. All authors contributed to the article and approved the submitted version.

## Funding

This research was supported by the National Natural Science Foundation of China under Grant No. 71764033.

## Conflict of Interest

The authors declare that the research was conducted in the absence of any commercial or financial relationships that could be construed as a potential conflict of interest.

## Publisher’s Note

All claims expressed in this article are solely those of the authors and do not necessarily represent those of their affiliated organizations, or those of the publisher, the editors and the reviewers. Any product that may be evaluated in this article, or claim that may be made by its manufacturer, is not guaranteed or endorsed by the publisher.
